# Validity and reliability of the Evidence Utilisation in Policymaking Measurement Tool (EUPMT)

**DOI:** 10.1186/s12961-017-0232-6

**Published:** 2017-08-04

**Authors:** M. H. Imani-Nasab, B. Yazdizadeh, M. Salehi, H. Seyedin, R. Majdzadeh

**Affiliations:** 1 Social Determinants of Health Research Center, Lorestan University of Medical Sciences, Khorramabad, Iran; 2 Department of Public Health, School of Health and Nutrition, Lorestan University of Medical Sciences, Khorramabad, Iran; 3 Knowledge Utilization Research Center, Tehran University of Medical Sciences, Tehran, Iran; 4 Department of Biostatistics, School of Public Health, Iran University of Medical Sciences, Tehran, Iran; 5 Department of Health Services Management, School of Health Management and Information Sciences, Iran University of Medical Sciences, Tehran, Iran; 6 Department of Epidemiology and Biostatistics, School of Public Health, Tehran University of Medical Sciences, Tehran, Iran

**Keywords:** Knowledge translation, Health policymaking, Confirmatory factor analysis, Theory of planned behaviour

## Abstract

**Background:**

As a well-known theory in studying the effective factors on behaviour, the theory of planned behaviour (TPB) is frequently used in evaluating the health behaviour of people and healthcare providers, but rarely applied in studying the behaviour of health policymakers. The aim of the present study is to design and validate a TPB-based measurement tool for evidence utilisation in health policymaking (the EUPMT) through a mixed approach using confirmatory factor analysis.

**Methods:**

The study population consisted of all the specialised units and their employees in the five deputies of Iran’s Ministry of Health and Medical Education in 2013. All those eligible were invited to participate in the study, which comprised 373 persons. The reliability of the EUPMT was determined through test-retest and internal consistency. Additionally, its validity was determined by face, content, convergent, discriminant and construct validities. SPSS-20 and LISREL-8.8 were employed to analyse the data. To assess the fitness of the measurement models, three groups of indices were used, i.e. absolute, relative and parsimonious.

**Results:**

The content and face validities of the tool were 83% and 67%, respectively. Cronbach’s alpha of different constructs ranged from 0.7 to 0.9. In the test-retest method, the intra-class correlations were between 0.75 and 0.87. Confirmatory factor analysis showed that the penta-factorial structure of the experimental data had acceptable fitness with the TPB (GFI = 0.86, NFI = 0.94, RSMEA = 0.075).

**Conclusion:**

TPB is able to explain the behaviour of evidence utilisation in health policymaking. The finalised TPB-based tool has relatively good reliability and validity to assess evidence utilisation in health policymaking. The EUPMT can be applied to determine the status quo of evidence utilisation in health policymaking, whilst designing interventions for its improvement and assessing their outcomes.

## Key message

- TPB is able to explain the behaviour of evidence utilisation in health policymaking.
- The finalised TPB-based tool has relatively good reliability and validity to assess evidence utilisation in health policymaking.
- The EUMPT can be applied to determine the status quo of evidence utilisation in health policymaking, whilst designing interventions for its improvement and assessing their outcomes.

## Background

Health systems often fail to meet the optimal utilisation of research evidence, which itself may lead to inefficacy, reduced quality of life, the life expectancy of citizens and, as a result, reduced productivity [1]. At an international level, there is an increasing interest in health system policymakers’ and managers’ awareness of relevant and valid research results [2–6]. Among the existing challenges is the difficulty in measuring policymakers’ research-use actions before and after the interventions aimed at promoting the practical utilisation of evidence [7, 8]. In such cases, we are left with no choice but to employ subjective methods, i.e. self-reporting of intention and/or behaviour, such as those derived from social cognition theories [9, 10].

**Fig. 1:**
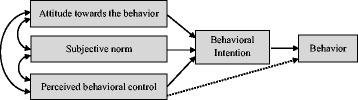
Theory of planned behaviour

To study behaviour-related factors, several theories have been presented, including Implementation Intention, Common Sense Self-regulation Model, Operant Learning Theory, Stage Model, and Theory of Planned Behaviour (TPB). TPB has been the dominant theoretical approach to study health-related behaviours over the past three decades [11]. This theory, originally presented by Ajzen [12], includes three constructs consisting of Attitude Towards to the Behaviour (ATB), Subjective Norm (SN) and Perceived Behavioural Control (PBC), which shape individuals’ intention, and intention is considered predictive of future behaviour. Figure 1 shows the conceptual model of TPB. TPB has been widely used to study health-related behaviours and is effective in predicting the individual health-related behaviours [13, 14].

TPB has been extensively applied in psychology and healthcare disciplines, and its efficacy in predicting individual health-related behaviour has been shown in a couple of systematic reviews [10]. Based on the results of systematic reviews conducted in psychology, this theory has been able to explain approximately 39% of changes in intention and 27% of changes in behaviour [15]. Moreover, existing evidence supports the use of TPB in predicting research evidence utilisation (clinical guidelines) by health specialists [10]. According to a systematic review [15], prediction of behaviour on the basis of health specialists’ intentions is similar to the values reported in other behaviour domains. Therefore, it may be helpful in studying the behaviour of health policymakers.

The application of TPB on any behaviour requires the identification of affecting factors on the intended behaviour and the development of a specific tool to measure their effects on the behaviour. Like any other measurement tool, an evaluation of the psychometric properties of this tool, such as validity and reliability, is required [10]. There are two approaches for designing a tool for the study of a behaviour based on TPB, namely the direct and indirect approaches. In the direct approach, the respondents’ overall judgment on the theory’s constructs is questioned. On the other hand, in the indirect approach, the respondents’ beliefs on the theory’s constructs are first questioned, and then the importance of the beliefs or the effects of them on the intention/behaviour are evaluated. The advantage of the indirect approach over the direct one is the possibility of accurately identifying the factors affecting intention and behaviour, and designing interventions to improve them [14]. The only developed TPB-based tool to measure the use of evidence in policymaking is that by Boyko et al. [10]. Nevertheless, due to the small sample size of the study, only its reliability was examined. Further, the approach taken by Boyko et al. [10] to design the questions was direct, which lacks the necessary efficacy to design knowledge translation interventions. Additionally, a protocol was published to study the factors affecting evidence utilisation for health policymaking based on TPB [7]. In this protocol, Lavis et al. [7] investigated the effects of access to a database of systematic reviews related to health policymaking on the intention of policy analysts and advisers in the Ministry of Health of Ontario, Canada, using the improved tool presented by Boyko et al. [10].

Makkar et al. [16], in a systematic literature review, found six developed tools to assess the utilisation of research evidence by policymakers. They highlighted the weaknesses of these tools, including not following a clear conceptual framework, assessing utilisation of research evidence of a particular policy or in general, not considering the critical appraisal of research evidence, assessing the use of research evidence in a long period of time and hence increasing the possibility of recall bias, not using the triangulation methods in data collection and, finally, not considering the imposed use of the research evidence. By developing the Staff Assessment of engagement with Evidence (SAGE) tool [16], they tried to overcome the limitations of previous tools and to objectively measure the utilisation of evidence in health policymaking.

Brennan et al. [17] developed and validated the Seeking, Engaging with and Evaluating Research (SEER) tool to measure the capacity and use of evidence by policymakers. This tool has three scales, namely those of individual capacity, research engagement actions, and the use of research. The subscales of individual capacity are similar to constructs of the Evidence Utilisation in Policymaking Measurement Tool (EUPMT), in as much as the individual values subscale is similar to the construct of ATB, the subscale of organisational values is similar to the SN construct, and the subscales of self-efficacy and organisational tools and systems are similar to the sub-constructs of PBC (self-efficacy and controllability, respectively). Another scale of the SEER tool involves research engagement actions that are similar to the behaviour construct of the EUMPT, but their questions are graded on two choices (Yes/No) rather than on a five-point Likert scale.

The reliability of the SEER tool was examined by test-retest and internal consistency and its validity was tested by confirmatory factor analysis and criterion validity. The bivariate correlations of individual capacity subscales and corresponding constructs of the tool proposed by Brennan et al. [17] present its criterion validity (0.419–0.671).

The present study was conducted to design a tool to assess evidence utilisation in health policymaking through the indirect approach to design predicting constructs of TPB and to assess its validity, reliability and factorial structure. Our aim was to design a tool that may be employed to determine the status quo (current beliefs and intentions about using research to inform policy), as well as to design interventions and assess their outcomes. The ultimate aim was to help promote the utilisation of evidence in health policies and its transformation into an organisational culture in health policymaking organisations.

## Methods

A cross-sectional study of factor analysis type was conducted to design and determine the validity, reliability and factorial structure of the tool evaluating evidence utilisation in health policymaking.

### Study population

The study population consisted of specialised units and their employees in the Central Department of Iran’s Ministry of Health and Medical Education (MOHME), including the office director, officer-in-charge, Head of the National Health Plan and expert officers (Table 1). The Central Department of MOHME comprises five deputies for Health Affairs, Curative Affairs, Nursing, Traditional Medicine, and Food and Drugs. Each deputy consists of a number of general offices, and each general office consists of a number of specialised units. Usually, the specialised units are where the policy briefs are developed.

**Table 1:** Characteristics of the participants in the construct validity test

Characteristics		Number	Percentage
Sex	Female	214	62
	Male	125	36
	No response	7	2
Age	20–30	13	4
	31–40	116	33.5
	41–50	177	51
	51–60	35	10
	No response	5	1
Professional experience	2–5	36	10
	5–15	117	34
	15–25	161	46.5
	25–30	27	8
	No response	5	1
Academic field	Clinical	113	33
	Para-clinical	60	17
	Health sciences	97	28
	Pharmacology	60	17
	Other	11	3
	No response	5	1
Level of education	Bachelors	57	17
	Masters	111	32
	Professional doctorate	100	29
	Doctor of Philosophy	73	21
	No response	5	1
Position	Officer	197	57
	Officer in-charge	80	23
	Office director	61	18
	Other	3	1
	No response	5	1
Place of work	Deputy of Health Affairs	189	55
	Deputy of Curative Affairs	64	18.5
	Deputy of Food & Drugs	78	22.5
	Deputy of Nursing	5	1
	Deputy of Traditional Medicine	5	1
	No response	5	1

### Sample size and selection

The inclusion criteria of the study were a minimum of 2 years’ professional experience in the Central Department of MOHME and participation in the development of policy briefs. Although some guidelines about sample size for confirmatory factor analysis (CFA) have been published so far, there is no consensus regarding this [18]. Many researchers recommend a minimum of 200 observations [19–21]. According to a rule of thumb, between 5 and 10 observations are required for every free parameter [22, 23]. Bearing in mind the 32 variables, a minimum of 160 samples was necessary. However, the number of eligible individuals was 373, which was about 12 times the number of the variables. Since the number of eligible individuals fell into the range of observations recommended for CFA, all of them were invited to participate in the study.

### Designing the questions

There are two approaches to designing the questions of predicting constructs of TPB (ATB, SN and PBC), namely the direct and indirect approaches [14]. In the direct approach, the overall judgment of the respondents is questioned about each theory construct, for example, to measure the ATB construct, some questions arise such as ‘the use of evidence in developing the health policy briefs is … (answer: five-point scale from helpful to unhelpful).

In the indirect approach, a qualitative study is first designed and implemented based on TPB constructs. To measure the construct of the ATB, some open questions arise, e.g. ‘in your opinion, what are the advantages of using evidence in developing the policy briefs?’ If one of the stated advantages is, for example, ‘to avoid wasting the resources’, to measure this attitude, two questions are designed as follows: 1. Developing the evidence-based policy briefs prevents wasting the resources (answer: five-point scale from likely to unlikely); 2. Preventing wasting the resources is … for me (answer: five-point scale from quite desirable to quite undesirable). As it might be noted, the first question measures the likelihood of studied behavioural outcomes while the second measures its utility in respondent opinion. The indirect approach is superior to the direct approach such that, in the indirect approach, specific factors related to the behaviour are identified and measured; therefore, using a tool that has been designed based on this approach can be more helpful in accurately designing the interventions for improving the considered behaviour.

The required qualitative study has already been carried out and published to identify the effective variables on the use of evidence in health policymaking based on TPB [24]. In this study, 32 variables were identified based on the indirect approach, where for each identified variable, two questions, and therefore a total of 64 questions, were designed. To design the questions related to intention and behaviour constructs, three and four questions were designed through a direct approach, respectively. Thus, the total number of questions in the initial tool was 71 (Table 2).

**Table 2:** Initial version of Evidence Utilisation in Policymaking Measurement Tool

#	Question	Construct	Validity & reliability^a^
1	Recall the last policy brief to which you contributed and assess it on the basis of the following respects: No search of papers and reports – comprehensive search of papers and reports	Behaviour	1
2	No attention to quality of papers – assessment quality by the critical appraisal tools	Behaviour	1
3	No use of opinions and experiences – use of all stakeholders’ opinions and experiences	Behaviour	1
4	No use of standard frameworks – use of the standard frameworks (1-3-25, policy brief)	Behaviour	3
5	I intend to develop the policy briefs on the basis of evidence	Intention	1
6	Development of policy briefs on the basis of evidence makes prevention of resources wastage	Attitude to behaviour	1
7	Prevention of the resource wastage is … to me		
8	The process of developing an evidence-based policy brief is time-consuming	Perceived behavioural control	5
9	The time-consuming nature of the process makes me give up developing the evidence-based policy brief		
10	Using the evidence helps in different steps of the policymaking cycle (prioritising the issues, identifying the most effective policies, implementing and evaluating)	Attitude to behaviour	2
11	Contributing to the different stages of the policymaking is … for me		
12	The policy briefs that develop on the basis of evidence do not adapt to the conditions in my country	Attitude to behaviour	4
13	The adaptation of policy briefs with the conditions of my country is … to me		
14	The development of evidence-based policy briefs disrupts the timely problem solving	Attitude to behaviour	1
15	Solving the problems on time is … to me		
16	Development of policy briefs on the basis of evidence improves the quality of our decisions	Attitude to behaviour	1
17	The improvement of the quality of our decisions is … to me		
18	Development of policy briefs on the basis of evidence will update my professional knowledge and will increase my creativity	Attitude to behaviour	1
19	The updating of professional knowledge and the increased creativity is … to me		
20	My superior expects me to develop the policy briefs on the basis of evidence	Subjective norm	1
21	The expectations of my superior are … to me		
22	The policymaking authorities (the health policy council, the board of deputies, and the board of directors) support the development of evidence-based policy briefs	Subjective norm	1
23	The support of policymaking authorities for the development of evidence-based policy briefs is … to me		
24	Deputy Minister of Health in my work area supports me in developing the evidence-based policy briefs	Subjective norm	4
25	The opinion of Deputy Minister of Health in my work area is … for me		
26	The administrators of policies (the medical science universities and the service providers) show interest in the evidence-based policy briefs	Subjective norm	1
27	The interest of the administrators of plans and policies is … to me		
28	The development of evidence-based policy briefs paves the way for the participation of other stakeholders (NGOs and other public organisations)	Subjective norm	1
29	The participation of other stakeholders in the policies is … to me		
30	Decisions regarding the scope of my work are made in critical condition	Perceived behavioural control	4
31	The critical conditions of decisions in the scope of my work make it … for me to develop the evidence-based policy briefs		
32	My colleagues develop the policy briefs on the basis of evidence	Subjective norm	5
33	Doing what my colleagues do is … to me		
34	I expect to develop the policy briefs on the basis of evidence	Intention	1
35	Programs/policies in my working field depend on the specific conditions such that the use of global evidence would be impossible	Perceived behavioural control	4
36	Using the global evidence in developing the policy briefs is … for me in the particular circumstances of our country		
37	For the development of evidence-based policy briefs, I have enough access to research evidence	Perceived behavioural control	4
38	My access to the required research evidence makes it … for me to develop evidence-based policy briefs		
39	My accessibility to the required routine data (organisational information and statistics) for the development of evidence-based policy briefs is limited	Perceived behavioural control	1
40	My accessibility to the routine data makes it … for me to develop the evidence-based policy briefs		
41	My relevant general director expected me to provide evidence-based policy briefs	Perceived behavioural control	1
42	Expectations of the general director are … for me		
43	My accessibility to the cost data (the cost of goods and services) for the development of evidence-based policy briefs is limited	Perceived behavioural control	5
44	My accessibility to the cost data makes it … for me to develop the evidence-based policy briefs		
45	For the development of evidence-based policy briefs, I do not have the possibility to use the opinions and experiences of different stakeholders (faculty members, service providers and receivers, and other organisations)	Perceived behavioural control	1
46	Inability to use the opinions and experiences of different stakeholders makes it … for me to develop the evidence-based policy briefs		
47	Most managers of the Headquarter of the Health Ministry are selected among the faculty members	Perceived behavioural control	5
48	The selection of the managers of the Headquarter of the Health Ministry among the faculty members … me to develop the policy briefs on the basis of evidence		
49	In addition to the evidence-based policy briefs, other factors (such as the pressure groups, the interests of the ruling coalition, lobbying, etc.) can influence the policies	Perceived behavioural control	1
50	Effects of other factors on policies … me from developing the evidence-based policy briefs		
51	The Headquarters of the Health Ministry suffers from management instability	Perceived behavioural control	1
52	Management instability makes it … for me to develop evidence-based policy briefs		
53	Experts of the Headquarters of the Health Ministry are not selected on the basis of their competencies in developing the evidence-based policy briefs	Perceived behavioural control	5
54	The selection of the experts of the Headquarters of the Health Ministry not on the basis of their competencies leads to the development of policy briefs not on the basis of evidence		
55	My spiritual copyright to develop the evidence-based policy briefs is not observed	Perceived behavioural control	4
56	Infringement of my spiritual copyright … me from developing the evidence-based policy briefs		
57	I want to develop the policy briefs on the basis of evidence	Intention	1
58	Even if policy briefs are not based on evidence, they will be discussed at the policymaking meetings, and they will have the chance of being adopted	Perceived behavioural control	1
59	The adoption of policy briefs which are not based on evidence … me from developing the evidence-based policy briefs		
60	My workload is heavy	Perceived behavioural control	5
61	My workload makes it … for me to develop evidence-based policy briefs		
62	My workplace is crowded and noisy	Perceived behavioural control	5
63	My crowded and noisy workplace makes it … for me to develop the evidence-based policy briefs		
64	The development of evidence-based policy briefs is not a standard for the measurement of my performance	Perceived behavioural control	1
65	Lack of assessment of my performance on this standard … me from developing the evidence-based policy briefs		
66	My salary payment is not based on the development of evidence-based policy briefs	Perceived behavioural control	1
67	This basis of payment … me from developing the evidence-based policy briefs		
68	Development of the evidence-based policy briefs is not a criterion for job promotion (in the organisational hierarchy) in the Health Ministry Headquarters	Perceived behavioural control	1
69	This promotion basis discourages me from developing the evidence-based policy briefs		
70	The Minister of Health supports me in developing the evidence-based policy briefs	Subjective norm	4
71	The opinion of the Minister of Health is … for me		

^a^Status of validity and reliability:

1. Approval of the validity and reliability

2. Disapproval of the content validity

3. Disapproval of the internal consistency

4. Disapproval of the test-retest reliability

5. Disapproval of the construct validity

### Ethical considerations

Before conducting the study, the necessary permissions were acquired from the Ethics Board of Iran University of Medical Sciences (IUMS: 93.105.352). To ensure the confidentiality of data, the completed questionnaires were received in sealed envelopes and opened on a certain day. Completion of the questionnaire was followed up three times. If lost, another questionnaire would be handed over to the participant. The researcher tried to gain the informed participation consent of the eligible individuals by introducing himself and explaining the aim and process of the study. In the case of expressing informed consent by participants, the questionnaires were given to them and a specific date was assigned for its delivery.

### Tool validity

The validity of the tool was tested through face, content and construct validities, and subsequently improved. Content validity refers to the capability of selected questions in reflecting features of measured constructs. Face validity assessed to what extent the questions of a scale are similar to the issue that they are designed to measure. Both validities can be calculated for any of the questions of a scale and to the whole scale [25–27]. Quantitative and qualitative methods were used to evaluate and improve the content and face validities. The designed tool was handed out to three groups of individuals, which were 18 members overall, including five content experts (people familiar with TPB), eight lay experts (evidence-based policy brief developers in the Central MOHME), and five methodologists (people who are experienced in questionnaire design and have published related articles). These individuals assessed the content and face validities of the questions and rated them in a varying degree of ‘completely desirable’ to ‘completely undesirable’. Then, the respondents were asked for their opinions on how the face validity of questions could be improved. The quantitative results of the test were estimated in the form of content validity ratio (CVR) and content validity index (CVI) indicators. CVR is calculated by dividing the number of individuals who believe the relevancy of the question is desirable and strongly desirable (in a four-point scale from ‘not desirable’ to ‘strongly desirable’) by the total number of respondents. The CVI is calculated by dividing the mean validity of each question by the total number of questions in the tool [25]. The questionnaire was revised by three panels of experts, including two methodologists, two topic experts, one Persian Language specialist, a MOHME expert, and our research team using the questions’ CVR, and suggestions were made regarding the omission or improvement of the questions’ validities.

Construct validity shows the extent to which observed scores on the tool are predicted by the theory upon which the tool was based. Accumulation of evidence about the face, convergent and discriminant validities of a tool is used to provide evidence of construct validity [28]. It is desirable that constructs are sufficiently correlated, but not so much that they cannot be discriminated from each other. Evidence of convergent validity can be demonstrated by showing a positive and significant correlation between scores for constructs that are expected to be related. The threshold considered as demonstrating convergent validity was 0.5. Discriminant validity is the extent to which a construct distinguishes itself from the other constructs. Exploratory Factor Analysis (EFA) and SPSS-20 software were used to test discriminant validity and CFA, while LISREL-8.8 was used to test convergent and construct validities. The Kaiser-Meyer-Olkin measure of sampling adequacy and Bartlett’s sphericity test were used to ensure the adequacy of the sample size and the existence of a correlation between the studied variables in the CFA. Before analysing the data with LISREL, the assumptions of the CFA were assessed for the existence of missing data, outliers, normality and linearity of the associations between the constructs [29, 30].

### Tool reliability

To evaluate the reliability of the direct questions, internal consistency was tested by calculating Cronbach’s alpha coefficient. Test-retest reliability of the indirect questions was tested by calculating the intraclass correlation coefficient (ICC) [14]. The time gap for the test-retest was approximately 2 weeks. The reliability test for the indirect questions was conducted in MOHME’s Deputy of Education; although it was not among the study’s target groups, it was very similar to the study population in terms of organisational settings. Next, 36 and 35 questionnaires were distributed in the test and retest phases, respectively. The response rates in the two phases were 94% and 91%, respectively.

To assess the goodness-of-fit of the measurement models, three groups of indices (absolute, relative and parsimonious) were used. Among the absolute fit indices, the χ^2^ to ‘degree of freedom’ ratio (acceptable < 3), the goodness-of-fit index (GFI) and the adjusted GFI (AGFI) (acceptable < 0.9) were used. The Bentler-Bonett normed fit index and the non-normed fit index were used from the relative fit indices, and the root mean squared error of approximation was employed as a parsimonious fit index. To ensure the accuracy of data entry, 10% of the data were imported to Excel software by another individual and the correspondence of data was evaluated with Epi-info software [31]. The discrepancy rate was less than 2%, which is acceptable. To examine the possibility of non-response bias, the characteristics of the individuals who refrained from completing the questionnaire, along with their reasons, were documented.

## Results

### Reliability

Upon testing the reliability of the indirect questions, the ICC of seven questions was lower than 0.6 and thus they were omitted along with their corresponding questions (questions: 12, 24, 30, 35, 41, 55 and 70). After removing these variables, the ICC of the behaviour, ATB, SN and PBC constructs, and that of the entire tool, were estimated to be 0.75, 0.83, 0.87 and 0.89, respectively. Cronbach’s alpha for the behaviour construct was smaller than 0.7, which improved after removing question 4. Cronbach’s alpha coefficients for the behaviour and intention constructs were 0.75 and 0.88, respectively.

### Validity

The response rate of the construct validity test was 92.76%. Despite following the participants up to three times and extending the dates of completion for the questionnaires, 27 individuals did not return their questionnaire. Non-response bias was not tested, as the response rate was high. Table 1 shows the characteristics of the participants in the construct validity test. Content validity of two questions (10 and 11) was not approved. The content and face validity indices of the tool and its constructs are presented in Table 3.

**Table 3:** The content and face validity indices of EUPMT

Constructs	Content validity index	Face validity index
Behaviour	0.82	0.68
Intention	0.80	0.74
Attitude	0.85	0.65
Subjective norm	0.81	0.80
Perceived behavioural control	0.80	0.76
Total	0.83	0.67

The Kaiser-Meyer-Olkin value was 0.89, indicating the adequacy of sample size for the CFA. The χ^2^ value calculated in Bartlett’s sphericity test was significant at *P* < 0.001, at a degree of freedom of 253, which is justified for the CFA test.

**Fig. 2:**
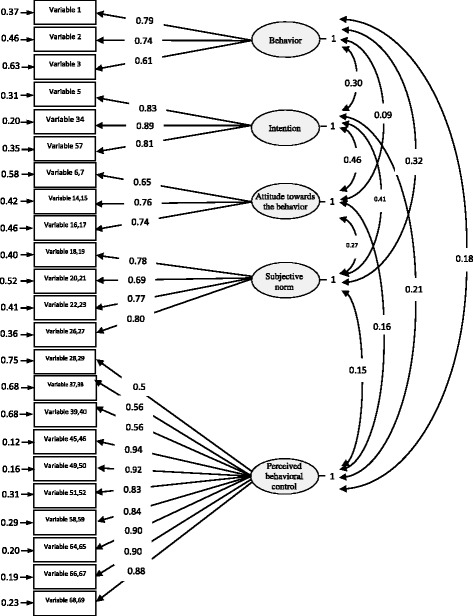
The measurement model of EUPMT for the standard estimate state

The TPB-based measurement model of EUPMT for the standard estimate state is illustrated in Fig. 2.

The results of the EFA revealed that most of the observed variables had a correlation higher than 0.5 with only one of the constructs, and their correlation with the other factors was lower than 0.5; hence, these results provided evidence of their discriminant validity. However, correlations predicted by the theoretical model for seven variables were not observed. The rotated factorial matrix output Varimax revealed six constructs instead of three. Upon comparing the factorial weight and the variables’ constructional associations, evidence of the discriminant validity of 17 variables was provided. One variable had no acceptable factorial weight in either of the constructs (questions 55 and 56). However, the remaining six variables may have represented the construct(s) that the theory was unable to explain. Thus, the status of these six variables was examined to see whether they could represent a new construct(s) or not. The remaining six variables were equally distributed among the three constructs supplementary to the theory. The variables ‘workload’ (Questions 60 and 61) and ‘crowded and noisy workplace’ (Questions 62 and 63) were placed in one construct. ‘The colleagues’ norm’ (Questions 32 and 33) and ‘selection of MOHME experts on the basis of their competencies’ were put in another construct. ‘Time-consuming nature of the process’ (Questions 8 and 9) and ‘selection of the managers of MOHME among the faculty members’ (Questions 47 and 48) were placed in the third construct.

The two variables ‘workload’ and ‘crowded and noisy workplace’ were evidently among the participants’ control beliefs and logically should have acquired suitable factorial weight in the PBC construct. Therefore, these variables were not able to build a new differentiated construct from the TPB constructs. ‘The colleagues’ norm’ and ‘selection of MOHME’ experts on the basis of their competencies’ did not have a mutual semantic association that could build a new distinctive construct from TPB constructs. Moreover, the former variable is evidently part of the SN construct, and the latter variable is part of the PBC construct. Therefore, logically, they should have gained good factorial weight in their corresponding constructs. The variables ‘time-consuming nature of the process’ and ‘selection of the managers of MOHME among the faculty members’ did not have a mutual semantic association such that they could form a new independent construct from TPB constructs. Furthermore, the participants considered ‘time-consuming nature of the process’ as a disadvantage of the evidence utilisation to develop a policy brief, which was a negative attitude toward the behaviour. Logically, it is expected that a good factorial weight will be found in its relevant construct, i.e. ATB. Most participants recognised ‘selection of the managers of MOHME among the faculty members’ as an environmental/organisational facilitator for developing evidence-based policy briefs, which is a positive control belief and should have acquired acceptable factorial weight in its relevant construct, i.e. PBC. Therefore, the surplus constructs of TPB derived from the EFA could not build new constructs to a better explanation for developing evidence-based policy briefs and thus they were removed along with their corresponding questions (questions 8, 32, 43, 47, 53, 60 and 62).

The factorial weight of the observed variables and the mean variance extracted for the other constructs was greater than 0.5; hence, these results provided evidence of their convergent validity. The mean extracted variance for each of the constructs was greater than the squared correlation coefficient (R^2^) of their variables with the other constructs; thus, these results provided evidence of their discriminant validity. Overall, evidence about the convergent and discriminant validity of 23 variables (40 questions) was provided.

Although the GFI and AGFI values slightly differ from the acceptable values (Table 4), the fitness indices give an acceptable fitness for EUPMT [29]. After refinement, the final version of the tool had 40 questions.

**Table 4:** Fitness indices for the assessment of the measurement model EUPMT

Index group	Index	Fitness	Acceptable fitness
Absolute fit indices	Goodness of Fit Index	0.86	˃ 90%
	Adjusted Goodness of Fit Index	0.82	˃ 90%
Relative fit indices	Normed Fit Index	0.94	˃ 90%
	Non-Normed Fit Index	0.95	˃ 90%
Parsimonious fit indices	Normed χ^2^	2.96	1–3
	Root Mean Squared Error of Approximation	0.075	˂ 0.08

## Discussion

### Fitness of the measurement model

The validity and reliability of EUPMT were examined using factor analysis, test-retest and internal consistency. The CVI of the tool was 83% and acceptable. The face validity of the tool was 67% and slightly smaller than the acceptable value; thus, it was improved in panels of experts. Upon testing the reliability of the directly observed variables via internal consistency, Cronbach’s alpha was between 0.7 and 0.9, and acceptable. Additionally, via testing of the reliability of the indirectly observed variables through test-retest, the ICC of different constructs was between 0.75 and 0.87, and the ICC of the tool was 0.89 overall and acceptable. The CFA results showed the acceptable goodness-of-fit indices for the measurement model of EUPMT.

To assess the validity and reliability of the SEER CFA, internal consistency and test-retest were used. The strength of the SEER was the approval of its criterion validity through simultaneous measuring with a TPB-based tool [17]; however, the criterion validity of EUPMT was not assessed. Another advantage of SEER is its consideration of the type of research usage (conceptual, instrumental, tactical and imposed). The results of CFA presented an acceptable fit of EUPMT and SEER tools. The reliability results of the test-retest of the constructs of EUMPT were superior to the corresponding subscales in SEER, such that ICCs of the constructs of ATB, SN and PBC sub-constructs (self-efficacy and controllability) were 16%, 7%, 2% and 17% higher than the corresponding subscales in SEER, respectively. The response rate in the examination of the validity and reliability of EUPMT was 92.8%, while this rate was 54%–55% in SEER.

Among the factors that lead to high rates of response in EUPMT study were the fame and reputation of one of the researchers (MR) and the fact that a signed invitation with the name of the participants was sent out; the measures to protect the confidentiality of responses (collecting the questionnaires in a sealed envelope and opening all of them on a given day); the interesting subject matter in the participants’ opinion; the timing delivery of completed questionnaires by participants within a 2-week period; three follow up reminders to receive the completed questionnaire and redelivering of the questionnaire in case of loss; collection of the data by the researcher instead of hiring interviewers; and suggesting the importance of the study considering the researcher’s degree (PhD), which was effective in motivating the participants. The researcher could draw the informed participation of the participants followed by introducing himself and explaining the objectives and steps of the study. Explaining the steps of the study was effective in increasing motivation. Finally, the fact that the questionnaires were not provided through translation or reviewing of previous texts, but rather were prepared through in-depth interviews of the participants’ co-workers, was also viewed positively.

EUPMT has been designed based on one of the most famous and prestigious behaviour theories (TPB). Moreover, SEER used the framework of SPIRIT (Supporting Policy In health with Research), which was developed based on a literature review and interviews with policymakers [32]. While the effectiveness of interventions based on the TPB has been demonstrated in different studies, the present study suggests the design and implementation of positive interventions for the use of evidence in health policymaking based on SEER and EUPMT and compares their effectiveness.

The reliability of the tool proposed by Boyko et al. [10] (assessing the intent of health policymakers to use evidence based on TPB) was also investigated through internal consistency (Cronbach’s alpha coefficients and generalisability) and test-retest reliability (Pearson correlation coefficients and generalisability); however, due to the small sample size (62 people), its construct validity was not tested using CFA. The alpha coefficient range of the constructs in Boyko et al.’s [10] tool was within 0.68 to 0.89, which was slightly lower than that of EUPMT (0.72 to 0.91). The advantage of EUPMT compared to the Boyko et al. [10] tool was to assess the construct validity through CFA. Another advantage of EUPMT was to use the indirect approach to design questions and hence the possibility of designing more specific and precise interventions to improve the use of evidence in health policymaking.

Among the limitations of the study is the use of self-reported data. To prevent social desirability bias, the questionnaires were received anonymously and in sealed envelopes. However, the bias may still be affecting the data. Another limitation of the study is the lack of testing of the criterion validity, which could provide further evidence of the validity of the EUPMT. Although the use of objective tools such as SAGE provides a more detailed profile of using the evidence in health policymaking, its use is difficult in practice. Other limitations of SAGE may be neglecting the mechanisms, structures and systems that can help improve the capacity of policymakers to use evidence, as well as focusing on barriers in using the evidence and ignoring the facilitators [16].

## Conclusion

The results showed that real data on utilisation of evidence in health policymaking support the TPB; in other words, the TPB is also capable of explaining the use of evidence in health policymaking. EUPMT has relatively good reliability and validity to assess evidence utilisation in health policymaking. This tool may be employed to determine the status quo of evidence utilisation in health policymaking, to design interventions for its improvement and to assess the outcomes of conducted interventions. The EUPMT can effectively help health policymakers promote the utilisation of evidence in the development of policy briefs and transform it into an organisational culture.

## Abbreviations

AGFI: Adjusted goodness-of-fit index
ATB: Attitude towards to the behaviour
CFA: Confirmatory factor analysis
CVI: Content validity index
CVR: Content validity ratio
EFA: Exploratory factor analysis
EUPMT: Evidence utilisation in policymaking measurement tool
GFI: Goodness-of-fit index
ICC: Intra-class correlation
MOHME: Ministry of Health and Medical Education
PBC: Perceived behavioural control
SAGE: Staff Assessment of engagement with Evidence
SEER: Seeking, Engaging with and Evaluating Research
SN: Subjective norm
SPIRIT: Supporting Policy In health with Research
TPB: Theory of planned behaviour
